# Hepatocellular Carcinoma in a non-Cirrhotic Liver of a HCV-Positive Woman with Sustained Viral Response

**DOI:** 10.4084/MJHID.2011.050

**Published:** 2011-10-26

**Authors:** L. Nosotti, T. D’Arca, M. Marignani, G. Balducci

**Affiliations:** 1 National Institute of Health, Migration and Poverty, Rome, Italy; 2 S. Andrea Hospital, Rome, Italy

## Dear Editor

We read with interest the recent review by Granito and Bolondi published in your journal.^1^

We appreciate in particular the consideration of the emerging role of metabolic syndrome as an important risk factor for hepatocellular carcinoma (HCC) development, and we describe a case of HCC developing in an African women without cirrhosis who achieved sustained viral response (SVR) after antiviral treatment for HCV-related chronic hepatitis. In this patient the main known risk factors for HCC development (high-grade fibrosis, overt/occult HBV infection, alcohol use, advanced age and male gender) were absent, and the only risk factor was diabetes.

## Case description

In march 2006, T.A., a 55-years-old Ethiopian woman, was diagnosed with HCV-correlated chronic hepatitis, genotype 1b, high viral load (quantitative HCV-RNA > 5,500,000 UI/ml) and slight serum aminotransferases increase (alanine aminotransferase (ALT) 46, aspartate aminotransferase (AST) 44; normal values: 6-31 IU/ml).

Liver ultrasound did not reveal neither focal echo-structural alterations, nor signs of portal hypertension and cirrhosis.

HCV infection duration was unknown and the patient was drug-naïve. The patient denied drug and alcohol use, blood transfusions and did not have piercings and tattoos. She was affected by diabetes mellitus type 2, and was under treatment with oral hypoglycemic drugs with good metabolic control. Schistosomiasis and HBV serology were negative, alpha-fetoprotein level was normal, and there were non contraindication to antiviral treatment. The patient refused liver biopsy.

Therefore, treatment with peg-IFN alpha 2a 180 mcg SC once weekly plus Ribavirin (RBV) 1000 mg/d PO was initiated. Rapid virological response (RVR) was not obtained at fourth week, but she reached early virological response (EVR) at week 12, and she successfully completed the scheduled 48 weeks treatment protocol. End of treatment response (ETR) was obtained at week 48, and sustained virological response (SVR) confirmed at week 24 follow up control visit (April 2008).

The patient did not attend further outpatient visit until January 2010, when ultrasound scan showed a 5-cm-diameter focal lesion suspicious for hepatic neoplasia. Abdominal CT scan confirmed the solid lesion (liver segments VIII and VII), which showed marked and early enhancement after contrast medium infusion, with peripheral ring and hyperdense star-shaped nucleus, dimension 5 × 4.5 cm, with associated altered vascularisation in the parenchymal area. The clinical report required liver biopsy to differentia the between focal nodular hyperplasia (FNH) and HCC.

On January 22^nd^, 2010, liver biopsy was performed and histological report described slight chronic hepatitis (G2 F1 according to Ishak score), and evidence of trabecular neoplasia with positive immunohistochemical markers consistent with the diagnosis of HCC.

Immunohistochemical staining for hepatitis B virus antigen (HBsAg) and core antigen (HBcAg) were negative.

On February 23^rd^, 2010, the patient firs underwent arterial chemoembolisation (lipiodol plus farmorubicin) of the HCC and later portal chemoembolisation.

On April 1^st^, 2010, follow up CT scan showed compensatory hypertrophy of the left liver lobe and successful chemoembolization outcomes, with hypodense areas near the main lesion attributable to necrosis of the hepatic tissue adjacent to the treated neoplasia.

On April 23^rd^, 2010, right hepatectomy was performed. Histological examination of the surgical sample showed poorly differentiated liver cancer with pleomorphic cytological features (G3) in the framework of slight chronic hepatitis with extensive necrotic areas. The neoplasm did not infiltrate resection margin. Post-operative course was uneventful.

In June 2010, abdominal CT scan showed right hepatectomy outcomes with regularly-bordered residual liver, without diffuse or focal alterations of the liver.

In October 2010, ultrasound scan evidenced further compensatory hypertrophy of the left lobe and no focal lesion.

Abdominal nuclear magnetic resonance performed in January 2011 was negative for focal lesions.

## Discussion

Some similar cases describing the occurrence of HCC in patients with chronic hepatitis C and SVR after interferon therapy have been reported in the literature.^2^-^8^

Makijama et al.^9^ demonstrated that risk factors for developing HCC in sustained responders after interferon therapy were: advanced age, male gender and more advanced histologic disease stage.

Iwasaki et al.^10^ observed that in patients with sustained viral response to interferon treatment, the main risk factors for HCC were the stage of fibrosis, age and alcohol abuse.

Another study^11^ on 1056 sustained responders showed that older age, higher serum AST level and lower platelet count before interferon therapy were independent risk factors associated with the development of HCC.

Ikeda et al.^12^ showed that in patients with chronic HCV infection responders to interferon therapy, an important risk factor for liver cancer was the concomitant presence of occult HBV infection.

Our case differs from those we have summarized since our patient did not have recognized risk factors: she was female, not old, with low-grade liver fibrosis, no alcohol consumption, low serum AST levels <100 mg/dl, normal platelet count and no overt or occult HBV co-infection.

A primary point to be discussed is the pathogenesis of HCC. It is well known that most HCCs develop in cirrhotic liver, but in 15-20% of cases it can also develop in non-cirrhotic liver.^13^ Therefore, our observation supports the notion that HCC may also develop in patients with chronic hepatitis C with mild fibrosis, as it happens in the case of HBV infection, where cirrhosis does not represent a pre-requisite for neoplasm formation.

HCC risk factors, besides HBV and HCV infection, include alcohol abuse, ingestion of genotoxic substances such as aflatoxins, smoking, metabolic and congenital disorders, sexual hormones therapy, iron overload, family case history of liver neoplasm and diabetes.^13^-^16^ In the case we have presented, apart from HCV infection, the only possible risks factors were aflatoxins exposition, which is frequent in Africa and Asia^15^ and diabetes.^16^

In particular, in the study of Hung et al,^16^ type 2 diabetes was an independent predictor of HCC in the subgroup of patients without cirrhosis who achieved SVR, and this finding is consistent with two other recent studies.^17^-^18^

Further studies are needed to better clarify the pathogenic mechanism of hepatocarcinogenesis in diabetic patients without cirrhosis after eradication of HCV.

## Conclusion

It has recently been demonstrated that also non-cirrhotic patients affected by chronic hepatitis C with SVR after antiviral therapy may be at risk for HCC, even if they show very mild fibrosis, and also after many years from SVR.^13^

In addition, our case confirm the need for a prolonged surveillance of non-cirrhotic patients even if they are sustained responders to antiviral therapy, although the exact incidence of HCC in these subjects is still unknown.
